# Dual-Emission Au-Ag Nanoclusters with Enhanced Photoluminescence and Thermal Sensitivity for Intracellular Ratiometric Nanothermometry

**DOI:** 10.3390/bios15080510

**Published:** 2025-08-06

**Authors:** Helin Liu, Zhongliang Zhou, Zhiwei Wang, Jianhai Wang, Yu Wang, Lu Huang, Tianhuan Guo, Rongcheng Han, Yuqiang Jiang

**Affiliations:** 1 Liver Research Center, Beijing Friendship Hospital, Capital Medical University, Beijing 100050, China; godlin@pku.org.cn; 2 State Key Laboratory of Digestive Health, Beijing 100050, China; 3 National Clinical Research Center for Digestive Diseases, Beijing 100050, China; 4 Institute of Genetics and Developmental Biology, Chinese Academy of Sciences, Beijing 100101, China; zhouzhongliang@genetics.ac.cn (Z.Z.); 15004044846@163.com (Z.W.); yuwang@genetics.ac.cn (Y.W.); huanglu@genetics.ac.cn (L.H.); 5 Single-Molecule and Nanobiology Laboratory, Department of Biochemistry and Biophysics, School of Basic Medical Sciences, Peking University, Beijing 100083, China; pistol@bjmu.edu.cn; 6 China Union of Life Science Societies, Beijing 100101, China

**Keywords:** bimetallic nanoclusters, intracellular temperature sensing, ratiometric nanothermometer

## Abstract

We report the development of highly luminescent, bovine serum albumin (BSA)-stabilized gold–silver bimetallic nanoclusters (Au-AgNCs@BSA) as a novel platform for high-sensitivity, ratiometric intracellular temperature sensing. Precise and non-invasive temperature sensing at the nanoscale is crucial for applications ranging from intracellular thermogenesis monitoring to localized hyperthermia therapies. Traditional luminescent thermometric platforms often suffer from limitations such as high cytotoxicity and low photostability. Here, we synthesized Au-AgNCs@BSA via a one-pot aqueous reaction, achieving significantly enhanced photoluminescence quantum yields (PL QYs, up to 18%) and superior thermal responsiveness compared to monometallic counterparts. The dual-emissive Au-AgNCs@BSA exhibit a linear ratiometric fluorescence response to temperature fluctuations within the physiological range (20–50 °C), enabling accurate and concentration-independent thermometry in live cells. Time-resolved PL and Arrhenius analyses reveal two distinct emissive states and a high thermal activation energy (*E_a_* = 199 meV), indicating strong temperature dependence. Silver doping increases radiative decay rates while maintaining low non-radiative losses, thus amplifying fluorescence intensity and thermal sensitivity. Owing to their small size, excellent photostability, and low cytotoxicity, these nanoclusters were applied to non-invasive intracellular temperature mapping, presenting a promising luminescent nanothermometer for real-time cellular thermogenesis monitoring and advanced bioimaging applications.

## 1. Introduction

Precise and non-invasive temperature sensing at the micro/nanoscale is of critical importance in fields ranging from intracellular thermogenesis monitoring to smart diagnostics and localized hyperthermia therapies [1,2,3,4,5]. Traditional luminescent thermometric platforms, such as rare-earth doped materials [6,7,8,9,10,11,12,13,14,15,16], quantum dots (QDs) [17,18,19,20,21], organic fluorophores [22,23,24], and polymer-based nanostructures [25,26], have been extensively explored for this purpose [27,28,29]. However, these systems often suffer from intrinsic limitations including high cytotoxicity, large particle size, low photostability, and environmental sensitivity (e.g., to pH or ionic strength) [30,31], which hinder their practical application in live-cell temperature mapping. 

In recent years, noble metal nanoclusters (NCs), particularly gold (Au) and silver (Ag) nanoclusters, have emerged as a promising class of fluorescent nanothermometers [32,33,34,35,36,37]. Comprising only a few to several dozen metal atoms, these nanoclusters generally possess dimensions smaller than 2 nm, quantum confinement effects, and molecule-like discrete electronic states, resulting in tunable PL, superior aqueous dispersibility, and excellent biocompatibility [38,39,40]. For example, bovine serum albumin (BSA)-protected Au nanoclusters (AuNCs@BSA) have been reported to exhibit temperature-sensitive emission and have been applied in aqueous nanothermometry [41]. Similarly, Ag nanoclusters stabilized by biomolecules such as peptide have also demonstrated potential in temperature sensing [42].

Despite these advances, the PL QY and temperature sensitivity of monometallic NCs remain suboptimal for high-contrast imaging and real-time intracellular sensing, typically exhibiting a QY < 10% and low thermal responsiveness. Therefore, engineering the photophysical properties of metal NCs via metallic doping has gained attention as a strategy to enhance their performance. Silver doping in gold nanoclusters (AuNCs) has been shown to significantly enhance their PL efficiency. For instance, a 3.5-fold increase in QY was reported for Au_25_Ag_2_ compared to its undoped counterpart, while another study observed a 3- to 5-fold enhancement in glutathione (GSH)-stabilized Au-AgNCs (Au-AgNCs@GSH) relative to AuNCs@GSH [43]. These improvements have been attributed to several factors, including modifications in electronic structure, the emergence of new luminescent centers within the bimetallic core, alterations in core size and morphology, and strengthened interactions between the metal core and stabilizing ligands. Nevertheless, it remains unclear whether silver doping can also enhance the temperature-sensing sensitivity of alloy nanoclusters, which is critical for their application in nanoscale thermometry.

Herein, we report a facile one-pot synthesis of BSA-stabilized Au-Ag bimetallic nanoclusters (Au-AgNCs@BSA) with significantly enhanced PL and temperature sensitivity. The introduction of Ag atoms into the gold core leads to a marked increase in PL QY (up to 18%) and improved thermal activation energy compared to pure AuNCs. Notably, these Au-AgNCs@BSA exhibit dual-emission characteristics with a ratiometric response to temperature changes, effectively eliminating the influence of probe concentration or excitation inhomogeneity—common challenges in single-emission-based thermometry [44].

Time-resolved PL and Arrhenius-type thermal quenching analyses further reveal distinct emissive states and mechanisms associated with ligand-to-metal charge transfer (LMCT) and metal-core transitions. Owing to their ultrasmall size, biocompatibility, and robust thermal response, these Au-AgNCs@BSA nanothermometers demonstrate great potential for accurate, real-time intracellular temperature sensing.

## 2. Materials and Methods

### 2.1. Materials

Hydrogen tetrachloroaurate(III) trihydrate (HAuCl_4_·3H_2_O), silver nitrate (AgNO_3_), and bovine serum albumin (BSA, ≥98%) were purchased from Sigma-Aldrich (Shanghai, China) and used as received. Rhodamine 6G was used as a fluorescence quantum yield reference standard. All other chemicals were of analytical grade and used without further purification. Deionized water (18.2 MΩ·cm) was used throughout all experiments. HeLa cells were cultured in Dulbecco’s Modified Eagle Medium (DMEM) supplemented with 10% fetal bovine serum and 1% penicillin–streptomycin.

### 2.2. Synthesis of Au-AgNCs@BSA

The Au-Ag bimetallic nanoclusters were synthesized via a one-pot aqueous method. In a typical procedure, 5 mL of BSA solution (50 mg/mL) was mixed with 5 mL of HAuCl_4_ aqueous solution (10 mM). Subsequently, an appropriate volume of AgNO_3_ solution was added to achieve a desired Au:Ag molar ratio (e.g., 19:1 for optimal luminescence). The mixture was stirred (500 rpm) at 37 °C for 5 min. Then, the pH was adjusted to 10.0 using 1.0 M NaOH, and the solution was incubated at 37 °C for 12 h to facilitate nanocluster formation. After that, ultrafiltrate the solution three times using an ultrafiltration tube with a 30 KD specification, and store the product in a sealed container in a refrigerator at 4 °C.

For comparison, monometallic AuNCs@BSA were synthesized under identical conditions without the addition of AgNO_3_.

### 2.3. Optical Characterization

UV-Vis absorption spectra were recorded using a spectrophotometer (Puxi, TU-1901, Beijing, China). Fluorescence emission and excitation spectra were obtained with the Hitachi F-4500 Fluorescence Spectrophotometer. The effects of photostability and biochemistry (pH and ion strength) on the PL of the Au-AgNCs@BSA were also examined using the Hitachi F-4500 Fluorescence Spectrophotometer. Fluorescence QYs were calculated relative to Rhodamine 6G (QY = 95% in ethanol) using standard comparative methods. The lifetime measurements of all samples were tested by a lifetime and steady-state spectrometer (Edinburgh Instruments Ltd. FLS980, Livingston, UK). The TEM analysis was performed on a JEOL F200 instrument at an acceleration voltage of 200 kV.

### 2.4. Temperature-Dependent Fluorescence Measurements

To evaluate the thermal sensitivity of the nanoclusters, fluorescence emission spectra were collected at various temperatures ranging from 20 to 45 °C using a temperature-controlled quartz cuvette holder (DC0506, Noki Instrument, Changzhou, China), with a temperature range of −5 to 100 °C and a temperature fluctuation of ±0.05 °C (at 25 °C with a medium of water or alcohol). Each sample was equilibrated at the target temperature for 5 min before measurement. The fluorescence intensity at 613 nm (and secondary peak, if applicable) was monitored as a function of temperature. Activation energy (*E_a_*) for thermal quenching was determined by fitting the data using the Arrhenius equation.

The ratiometric temperature-sensing performance was evaluated by calculating the fluorescence intensity ratio of dual emission peaks (*I*_1_*I*_2_) and determining its linearity and relative sensitivity (%/°C).

### 2.5. Cytotoxicity Assay

The cytotoxicity of the Au-AgNCs@BSA was assessed using the standard MTT assay. The HeLa cell line was obtained from the Cell Resource Center, Peking Union Medical College (which is part of the National Science and Technology Infrastructure, the National Biomedical Cell-Line Resource, NSTI-BMCR; http://cellresource.cn). HeLa cells were incubated with various concentrations of nanoclusters (0–200 µg/mL) for 24 h. Cell viability was quantified by measuring absorbance at 570 nm using a microplate reader, and viability was expressed as a percentage relative to untreated controls.

### 2.6. Cellular Uptake and Intracellular Localization of Au-AgNCs@BSA

To study the cellular uptake and intracellular localization of Au-AgNC@BSA, HeLa cells were firstly seeded at a density of 2000 cells/cm^2^ on a 20 mm confocal dish supplemented with FDS (10% FBS, 89% DMEM, and 1% PS). The cells were then cultured at 37 °C and 5% CO_2_ for 12 h, after which the culture medium was changed to DMEM for a further 5 h. The cells were then washed three times with a PBS solution (pH 7.4) and incubated with a mixture of 25 µL of Au-AgNC@BSA (1.0 mg/mL) and 175 µL of DMEM at 37 °C for 2 h. Finally, the cells were washed three times with a PBS solution, after which 2 mL of PBS was added for laser confocal imaging using a Zeiss 980 optical system.

## 3. Results and Discussion

### 3.1. Synthesis and Characterization of Au-AgNCs@BSA

**Figure 1:**
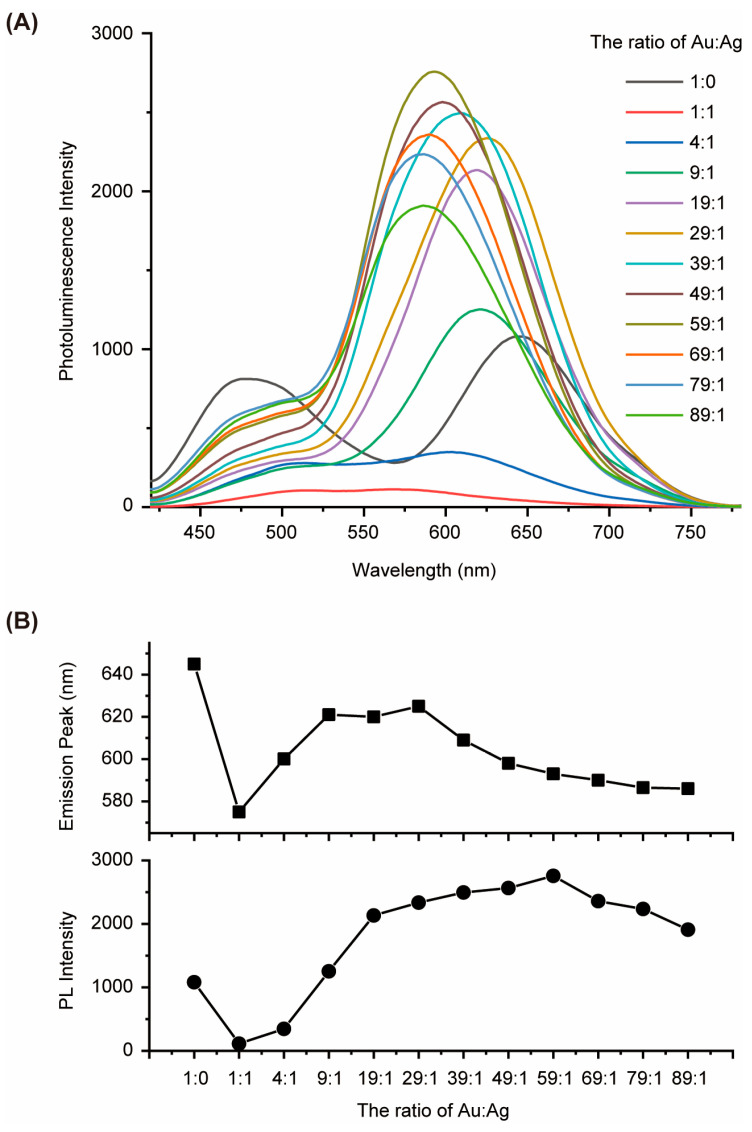
(**A**) PL spectra of Au-AgNCs@BSA with different Ag contents. (**B**) PL peaks and the PL intensities as a function of the ratio of Au:Ag.

The Au-Ag bimetallic nanoclusters were synthesized via a facile, one-step aqueous reaction using BSA as a stabilizing and reducing agent. By varying the molar ratio of Au^3+^ to Ag^+^ precursors, a series of nanoclusters was obtained with tunable emission properties. As shown in Figure 1A, the PL spectra of Au-AgNCs@BSA demonstrate significant shifts in emission peaks and changes in intensity as the silver content is adjusted. This tunability highlights the versatility of these nanoclusters, as their optical properties can be precisely controlled by simply varying the Au:Ag ratio. This feature is particularly advantageous for applications in sensing and imaging, where customizable emission profiles are highly desirable. The relationship between PL peak positions and intensities and the Au:Ag ratio is further elucidated (Figure 1B). The ability to fine-tune these properties by altering the metal composition underscores the potential of Au-AgNCs@BSA for a wide range of applications, including targeted imaging and highly specific sensing tasks. This tunability is a key innovation of our work, offering a flexible platform for optimizing the performance of these nanoclusters for various practical uses. Figure S1 illustrates that the Au-AgNC@BSA nanoparticles possess a spherical shape and have a size of 34.02 ± 11.45 nm (N = 2446), as determined by TEM analysis.

Further analysis indicates that appropriate silver doping can lead to enhanced PL QY. For instance, compared to monometallic AuNCs@BSA (QY 5.7%), Au-AgNCs@BSA exhibited significantly enhanced PL QY of 10% and 18% at Au:Ag ratios of 4:1 and 9:1, respectively, using Rhodamine 6G as a reference (Table 1). A comparable enhancement effect has been exhibited by AuNCs@BSA, which selectively reduce Ag^+^ to yield highly luminescent Au-Ag alloy NCs [45].

**Figure 2:**
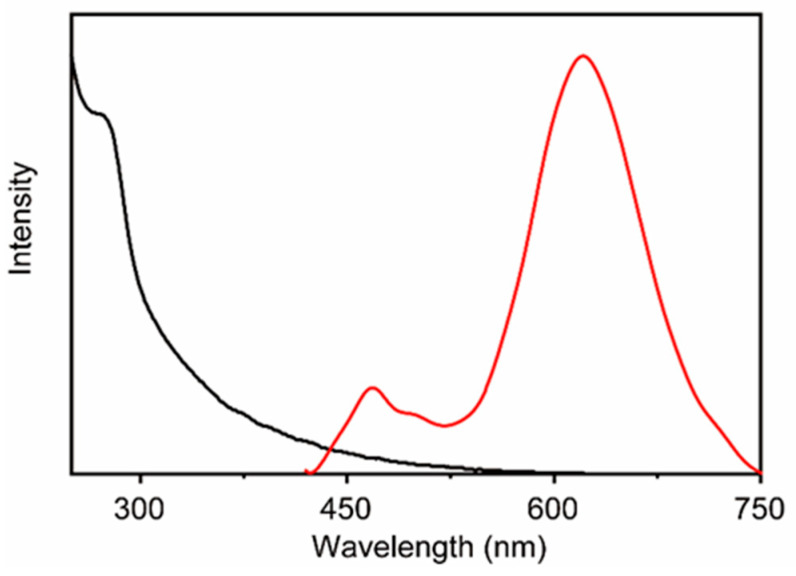
Representative absorption (black) and emission spectra (red) of Au-AgNCs@BSA.

The typical nanocluster, prepared at a Au:Ag molar ratio of 9:1 (QY 18%), exhibited a strong red emission centered at 613 nm under excitation at 365 nm (Figure 2). A weak blue fluorescence was observed at a wavelength of approximately 470 nm. This phenomenon could be attributed to the emission of BSA, as evidenced by the observation that pure BSA exhibits similar fluorescence in this region. This finding is consistent with previous reports [46]. 

Although the exact synthesis mechanism remains unclear, anti-galvanic reduction [47] is a likely process where metal ions (Ag^+^) are reduced by more noble metals within small gold nanoparticles. This technique has been used to offer a simple and gentle method for controlling the composition and structure of nanoscale alloys, which are typically difficult to manipulate. It is logical to assume that a similar process has taken place in our system. 

**Figure 3:**
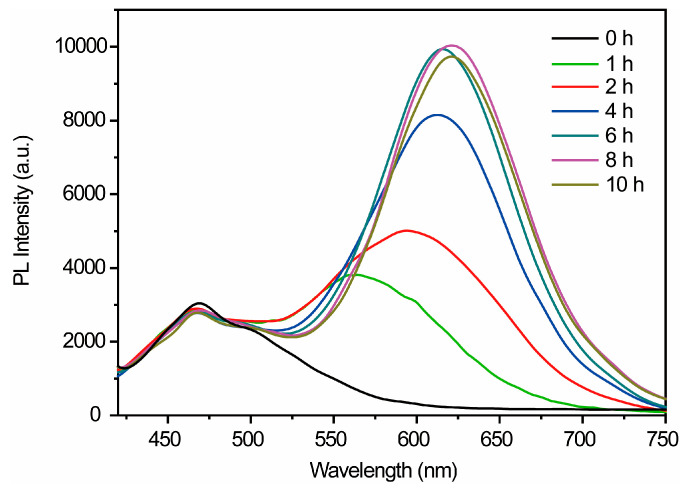
The dynamic PL spectra of Au-AgNCs@BSA as a function of increasing reaction time.

Furthermore, the dynamic PL spectra of Au-AgNCs@BSA were studied as a function of increasing reaction time (Figure 3). The PL spectra of Au-AgNCs@BSA evolve from blue (470 nm) to red (620 nm) over 10 h, reflecting changes in the coordination environment, core–shell structure, and Ag:Au ratio. BSA acts as a stabilizer, template, and ligand, influencing cluster formation and luminescence stability. Ag doping transforms the Au-dominated clusters into an alloy structure, shifting luminescence from charge transfer to metal–ligand mixed states. 

We propose that the synthesis comprises multiple steps, including metal ion reduction, nanocluster nucleation, growth, and rearrangement, as well as remodeling of the ligand metal core. Prolonged synthesis time stabilizes the clusters and boosts luminescence efficiency. The shift from blue to red emission indicates the transformation from unstable to stable clusters, representing a transition from no (or weak) luminescence to highly luminescent metal clusters.

### 3.2. Temperature-Dependent Emission Behavior of Au-AgNCs@BSA

**Figure 4:**
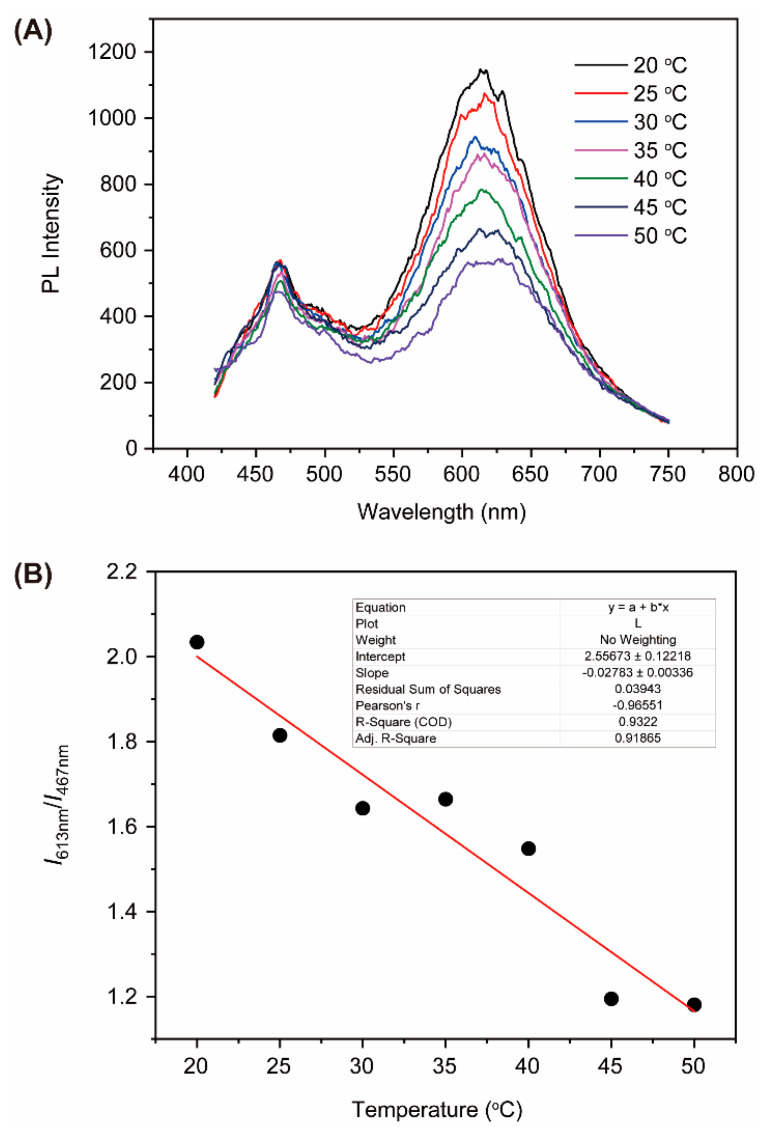
(**A**) PL spectra of Au-AgNCs@BSA under varying temperature conditions. (**B**) Ratio of PL intensity at 613 nm to that at 467 nm as a function of temperature.

The temperature sensitivity of Au-AgNCs@BSA was systematically investigated across a physiologically relevant range. Upon heating, the emission intensity at 613 nm decreased linearly, and the rate of decrease was significantly higher compared to AuNCs@BSA (Figure 4A). With an increasing temperature, the intensity of fluorescence at 612 nm exhibited a monotonous decrease, without no apparent emission peak shift. These phenomena are consistent with previous studies [48]. It is noteworthy that all the fluorescence properties are reversible in this temperature-dependent measurement, suggesting that no significant structural damage occurs during temperature changes. Moreover, while the emission spectra show marked temperature dependence, the absorption spectra remain virtually unaffected (Figure S2). This observation supports the notion that the transition between emissive states induced by temperature is primarily governed by excited-state dynamics rather than by changes in ground-state electronic configurations. This further confirms the structural integrity of the nanoclusters throughout the heating process.

While single-emission probes are often affected by probe concentration and excitation power fluctuations, Au-AgNCs@BSA showed dual emission peaks whose intensity ratio (*I*_1_/*I*_2_) varied linearly with temperature. This ratiometric response minimizes external interference and offers more accurate intracellular temperature measurement. As part of further analysis, the temperature-dependent PL behavior of Au-AgNC@BSA probes was evaluated by the intensity ratio of the 613 nm to 467 nm emission peaks. As shown in Figure 4B, the PL ratio increases linearly with increasing temperature from 20 to 50 °C. The response sensitivity is determined to be 1.39% per °C, which is better than the values of current ratiometric nanothermometers [49,50]. Assuming a precision of ±0.5% in the determination of the ﬂuorescence intensity, the resolution is about ±0.36 °C. This high temperature resolution is comparable to or better than the resolution of current fluorescent thermometers. Notably, the temperature resolution of our system is high enough for intracellular temperature mapping.

**Figure 5:**
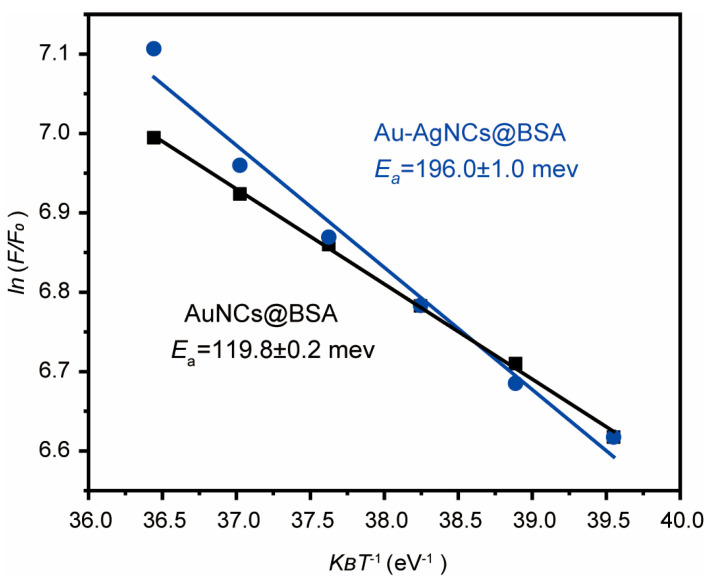
The Arrhenius plot of Au-AgNCs@BSA is pertinent to emission values at varying temperatures. For comparison, the Arrhenius plot of AuNCs@BSA is also presented. The values of activation energy (*E_a_*) are also indicated.

Further analysis indicated that the thermal quenching behavior followed an Arrhenius-type model:

(1) $$\frac{1}{F(T)}\;\;=\;\frac{1}{F_{0}}\;+A\exp(\frac{-E_{a}}{k_{B}T})$$

where *F*(*T*) is the fluorescence intensity at temperature *T*, *E_a_* is the activation energy, and *k_B_* is Boltzmann’s constant. As shown in Figure 5, the fitted *E_a_* for Au-AgNCs@BSA was determined to be 199 ± 7 meV, significantly higher than that of AuNCs@BSA (119.8 ± 0.2 meV). 

For comparison, activation energies (*E_a_*) of 103.6 meV and 130 meV have been reported for histidine-protected Au_10_ nanoclusters [51] and lipoic acid-protected Au nanoclusters [52], respectively. The *E_a_* for Ag nanoclusters embedded in glass was found to be 118.1 meV [32]. Additionally, BSA-protected Au_25_ NCs showed an *E_a_* of 62.5 meV [46]. For carbon dots, *E_a_* values of 24.0 ± 3.2 meV and 42.7 ± 3.9 meV were obtained for band-I and band-II, respectively [53]. Our results also compare favorably with non-MNCs such as organosilane-functionalized carbon dots (*E_a_* = 59.5 meV), which were proposed as optical probes for cell imaging [52].

According to the Arrhenius equation, a higher activation energy (*E_a_*) means that the system requires more energy to overcome the energy barrier between states. While this suggests that the transition is harder at a fixed temperature, it also means that the rate of change is more sensitive to temperature. As temperature increases, systems with high *E_a_* show a more dramatic increase in the transition rate, resulting in a more pronounced change in fluorescence intensity. Therefore, although a high *E_a_* implies that more energy is needed for transitions, it also indicates greater temperature sensitivity, making such systems ideal for temperature-sensing applications. In other words, activation energy (*E_a_*) reflects the sensitivity of fluorescence intensity to temperature changes: a higher *E_a_* indicates a more pronounced change in fluorescence with temperature, while a lower *E_a_* suggests weaker temperature dependence.

The phenomenon of enhanced temperature sensitivity resulting from silver doping can be attributed to the introduction of novel energy states and the alteration of charge transfer dynamics. Consequently, this results in excited-state processes becoming more responsive to temperature fluctuations. Additionally, Ag^+^ ions modify the local electronic environment and enable multiple emission pathways, the populations of which shift with temperature. Consequently, these complex photophysical interactions render the overall luminescence more temperature-sensitive.

**Figure 6:**
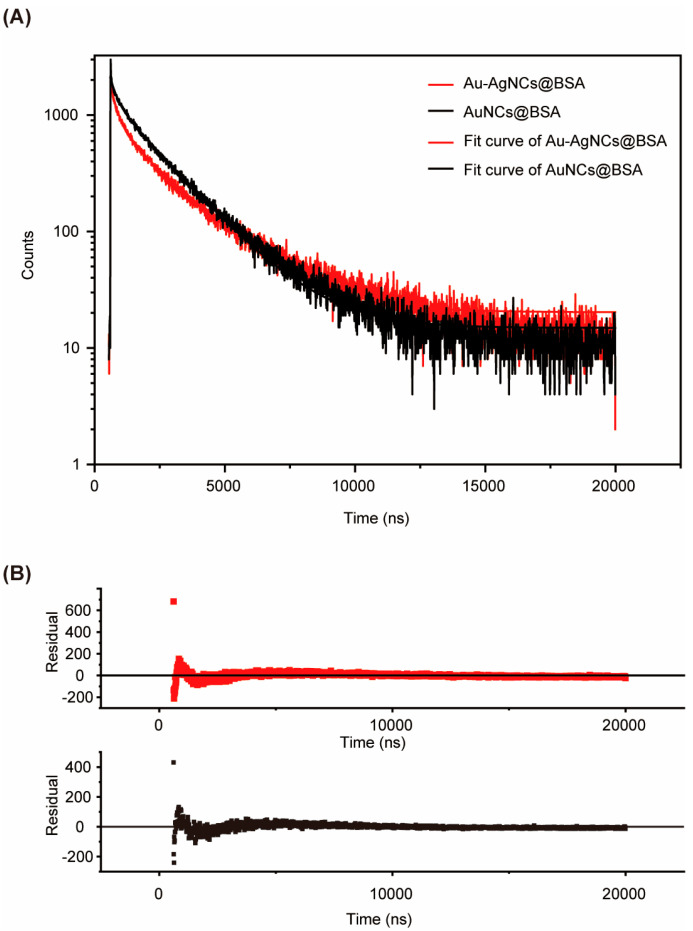
(**A**) Representative PL decays of Au-AgNCs@BSA in water. PL decay profiles were analyzed using a biexponential decay model. The characteristic decay times (*τ*_1_ and *τ*_2_, respectively) and their weights (*a*_1_ and *a*_2_) were obtained, as well as the mean PL lifetime (*τ*_mean_ = (*a*_1_*τ*_1_^2^ + *a*_2_*τ*_2_^2^)/(*a*_1_*τ*_1_ + *a*_2_*τ*_2_)). (**B**) The lower panels show residual differences between experimental and theoretical values.

To verify the hypothesis, the PL lifetime of the system was measured and the radiative/non-radiative transition rate was calculated. Time-resolved PL measurements revealed a biexponential decay profile for Au-AgNCs@BSA (Figure 6). The lifetimes (*τ*_1_, *τ*_2_) and their respective amplitudes correspond to two emissive pathways: a short-lived component attributed to intrinsic core-state emission; and a long-lived component associated with ligand-to-metal charge transfer (LMCT) and ligand-to-metal–metal charge transfer (LMMCT) [54,55]. Further analysis, as shown in Table 1, indicates that the average lifetimes of Au-AgNCs@BSA (*τ*_avg_ = 701.8 ns for Au:Ag = 1:0.11 and 778.3 ns for Au:Ag = 4:1) are notably shorter than that of pure AuNCs@BSA (*τ*_avg_ = 956.3 ns), indicating Ag-induced changes in electronic structure that accelerate radiative recombination. Further analysis indicates that Ag doping results in a significant increase in the radiative transition rate (*k_r_*), reaching 2.564 × 10^5^ s^−1^ at a 9:1 Au:Ag ratio—approximately 4.3 times higher than the undoped system—while the non-radiative rate (*k_nr_*) shows only a modest rise. This imbalance suggests that Ag primarily enhances fluorescence efficiency by promoting radiative decay pathways. Together, these changes in excited-state dynamics make Au-AgNCs@BSA highly effective as thermally responsive fluorescent probes.

### 3.3. Biochemical and Photostability Effects on PL of Au-AgNCs@BSA

**Figure 7:**
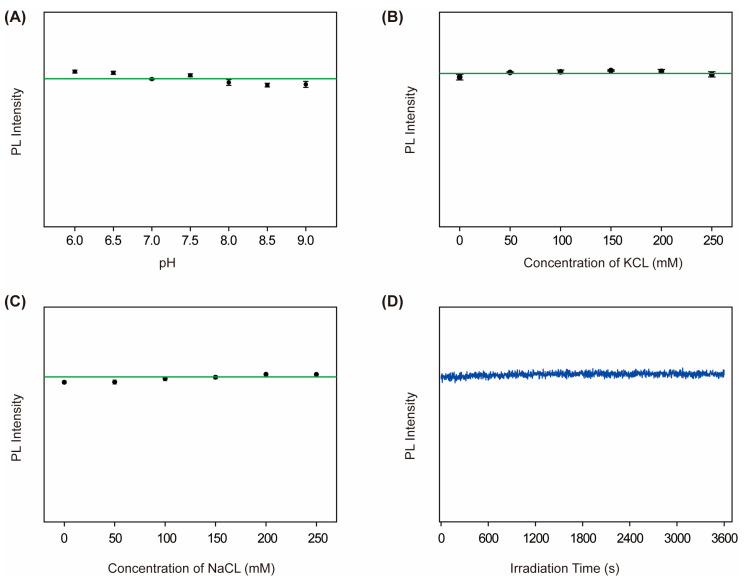
Effects of biochemical environmental factors such as pH, ionic strength, and prolonged light exposure on the PL intensity of Au-AgNCs@BSA. The PL intensity of Au-AgNCs@BSA is shown as a function of pH (**A**), KCl concentration (**B**), and NaCl concentration (**C**). (**D**) shows the PL intensities of Au-AgNCs@BSA as a function of irradiation time. For comparison, green horizontal auxiliary lines have been added to Figure 7A–C.

Owing to the intricate nature of the intracellular biochemical environment, fluorescence signals are vulnerable to the effects of pH, ionic strength, and photobleaching. A series of tests was conducted in order to evaluate the stability of Au-AgNCs@BSA under the aforementioned conditions. Initially, the pH stability was assessed by measuring the PL spectra of solutions with a pH range of 6.0 to 9.0. As demonstrated in Figure 7A, the PL intensity remained almost constant across the range of pH values examined, indicating optimal pH tolerance. Subsequently, the nanoparticles’ ionic stability was assessed by subjecting the nanoparticles to increasing concentrations (0–250 mM) of KCL and NaCL. As demonstrated in Figure 7B,C, the PL intensity exhibited negligible variation (<10%), indicating robust resistance to ionic interference. Consequently, photostability was assessed by subjecting the nanoparticles to continuous UV irradiation (365 nm, 700 V) for a duration of one hour. The time-course data (Figure 7D) demonstrated that there was no significant loss of PL, thereby confirming excellent long-term optical stability. These results collectively indicate that the PL of Au-AgNCs@BSA is highly stable under variable physiological and photonic conditions, thereby supporting their potential for reliable intracellular imaging and precise temperature detection.

### 3.4. Biocompatibility Evaluation for Biomedical Application

**Figure 8:**
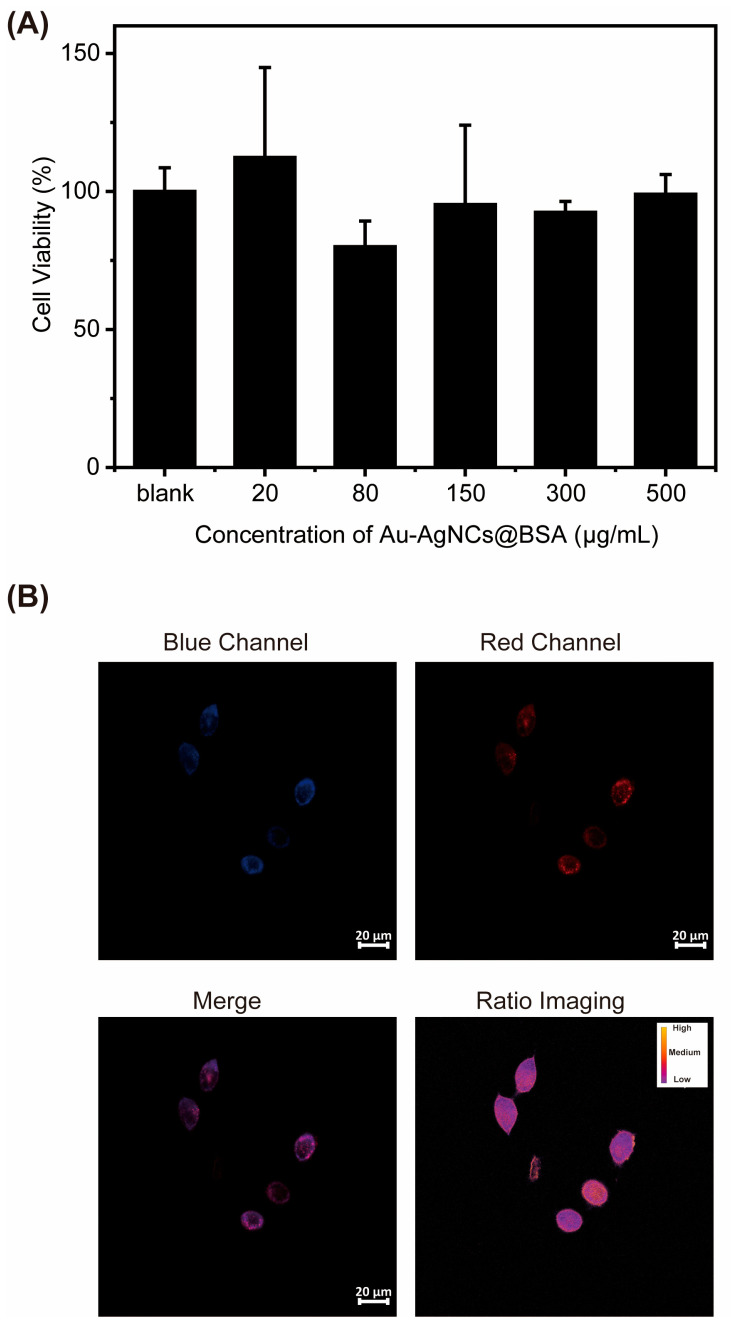
(**A**) MTT-based cell viability assay of HeLa cells after 24 h treatment with Au-AgNCs@BSA at varying concentrations of the nanoparticles. (**B**) The fluorescence microscopy images of HeLa cells pretreated with Au-AgNCs@BSA. The blue channel (408–552 nm), red channel (578–684 nm), and merged channel images, as well as the corresponding calculated ratio image of red to blue channels, are all shown.

For biomedical applications, biocompatibility is a critical consideration. In this study, the cytotoxicity of Au-AgNCs@BSA was assessed using HeLa cell lines. As shown in Figure 8, HeLa cells exhibited no significant adverse effects even at concentrations up to 500 μg/mL. The excellent biocompatibility of the material can be attributed to the intrinsic properties of gold and the shielding effect of BSA protein [56]. This superior property renders it highly favorable for biomedical applications, including long-term monitoring of intracellular temperature changes with minimal physiological disturbance.

Although our cytotoxicity assessment was limited to a 24 h incubation in HeLa cells, we recognize the importance of evaluating long-term biocompatibility and potential accumulation of the nanoclusters. For future in vivo applications, comprehensive studies on prolonged exposure, biodistribution, and clearance mechanisms—such as renal or hepatobiliary excretion—will be essential. Potential effects on major organs will also be examined via histopathological analysis and serum biochemical profiling in animal models.

Encouraged by the excellent biocompatibility and superior temperature detection performances of Au-AgNCs@BSA in solution, we evaluated the ability of Au-AgNCs@BSA in ratiometric imaging of intracellular temperature in HeLa cells. For this purpose, a blue channel (408–552 nm) and a red channel (578–684 nm) were set up for observation. As shown in Figure 8B, firstly, the blue channel and red channel emitted bright fluorescence when the HeLa cells were incubated in Au-AgNCs@BSA. Additionally, the merged channel image is presented, which combines the information from both the blue and red channels to provide a comprehensive view of the cellular fluorescence. Alongside these images, the corresponding calculated ratio image of the red to blue channel is also displayed. The ratio images with a pseudo-color calibration bar apparently displaye the heterogeneous temperature distribution within a cell. These cell imaging experiments collectively demonstrated that Au-AgNCs@BSA could serve as an efficient tool for ratiometric monitoring of intracellular temperature in living cells. Further colocalization experiments via 3D confocal fluorescence microscopy images of HeLa cells confirmed that Au-AgNCs@BSA accumulated mainly in the cytoplasm (Figure S3).

## 4. Conclusions

In summary, we have successfully developed a novel BSA-protected gold-silver bimetallic nanocluster (Au-AgNCs@BSA) system via a simple, one-step aqueous synthesis route. By fine-tuning the silver doping ratio, we achieved significantly enhanced PL properties, including a QY of up to 18%, and markedly improved temperature sensitivity. The incorporation of Ag atoms modulates the electronic structure of the nanoclusters, resulting in higher radiative decay rates and increased activation energy (*E_a_* = 199 meV), both of which contribute to superior thermal responsiveness.

Importantly, the dual-emission behavior of Au-AgNCs@BSA enables ratiometric temperature sensing, which effectively eliminates the influence of environmental factors such as probe concentration and excitation fluctuation. This property, combined with their small size, excellent water solubility, and low cytotoxicity, makes these nanoclusters highly suitable for real-time, non-invasive intracellular thermometry.

Our findings demonstrate that bimetallic doping is a powerful strategy to engineer the photophysical properties of noble metal nanoclusters for advanced sensing applications. The Au-AgNCs@BSA developed in this work represent a promising platform for luminescence-based thermal imaging and hold great potential for future applications in cellular metabolism monitoring, disease diagnostics, and intelligent nanomedicine.

## Figures and Tables

**Table 1:** The optical properties of Au-AgNCs@BSA and AuNCs@BSA.

The Ratio of Au:Ag	QY	*τ*_mean_ ^1^	*K*_r_ (×10^5^ s^−1^)	*K*_nr_ (×10^6^ s^−1^)
1:0	5.70%	956.3	0.596	0.986
9:1	18.0%	701.8	2.564	1.169
4:1	10.0%	778.3	1.284	1.156

^1^ *τ*_mean_ = (*a*_1_*τ*_1_^2^ + *a*_2_*τ*_2_^2^)/(*a*_1_*τ*_1_ + *a*_2_*τ*_2_)), where *τ*_1_ and *τ*_2_ represent short and long lifetimes, respectively, and *a*_1_ and *a*_2_ represent their respective weights.

## Data Availability

The original contributions presented in this study are included in the article. Further inquiries can be directed to the corresponding author.

## Supplementary Materials

The following supporting information can be downloaded at https://www.mdpi.com/article/10.3390/bios15080510/s1, Figure S1: Representative TEM image of Au-AgNC@BSA and the corresponding size distribution; Figure S2: The absorption spectra of Au-AgNCs@BSA at different temperatures; Figure S3: The fluorescence microscopy imaging of HeLa cells, pretreated with Au-AgNCs@BSA.

## Abbreviations

The following abbreviations are used in this manuscript:

BSA	Bovine serum albumin
QYs	Quantum yields
AuNCs	Gold nanoclusters
LMCT	Ligand-to-metal charge transfer
QDs	Quantum dots
