# Supplementary material for: Dual-Emission Au-Ag Nanoclusters with Enhanced Photoluminescence and Thermal Sensitivity for Intracellular Ratiometric Nanothermometry
Source: Biosensors (Basel). 2025 Aug 6;15(8):510. doi: 10.3390/bios15080510 (PMC12384061; doi:10.3390/bios15080510)
Supplement: Supplementary file 1 [file biosensors-15-00510-s001.zip › biosensors-3773359-supplementary.pdf]

Supplementary Materials

Dual-Emission Au-Ag Nanoclusters with Enhanced Photoluminescence and Thermal Sensitivity for Intracellular Ratiometric Nanothermometry

Helin Liu [1,2,3], Zhongliang Zhou [4], Zhiwei Wang [4], Jianhai Wang [5], Yu Wang [4], Lu Huang [4], Tianhuan Guo [6,*], Rongcheng Han [4,*] and Yuqiang Jiang [4,*]

1 Liver Research Center, Beijing Friendship Hospital, Capital Medical University, Beijing 100050, China; godlin@pku.org.cn
2 State Key Laboratory of Digestive Health, Beijing 100050, China
3 National Clinical Research Center for Digestive Diseases, Beijing 100050, China
4 Institute of Genetics and Developmental Biology, Chinese Academy of Sciences, Beijing 100101, China; zhouzhongliang@genetics.ac.cn (Z.Z.); 15004044846@163.com (Z.W.); yuwang@genetics.ac.cn (Y.W.); huanglu@genetics.ac.cn (L.H.)
5 Single-Molecule and Nanobiology Laboratory, Department of Biochemistry and Biophysics, School of Basic Medical Sciences, Peking University, Beijing 100083, China; pistol@bjmu.edu.cn
6 China Union of Life Science Societies, Beijing 100101, China
* Correspondence: guotianhuan@culss.org.cn (T.G.); hanrch@genetics.ac.cn (R.H.); yqjiang@genetics.ac.cn (Y.J.)

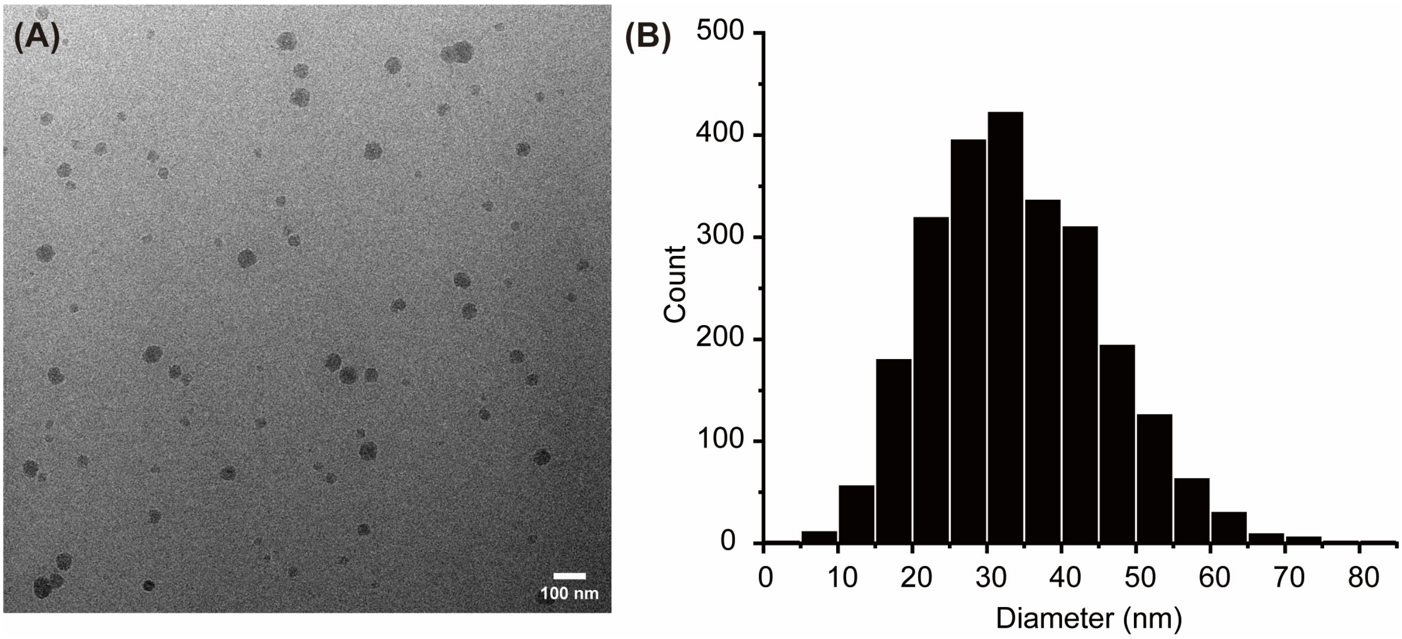

Figure S1. Representative TEM image of Au-AgNC@BSA and the corresponding size distribution. The diameter of these nanoparticles is determined to be 34.02 ± 11.45 nm (N = 2446).

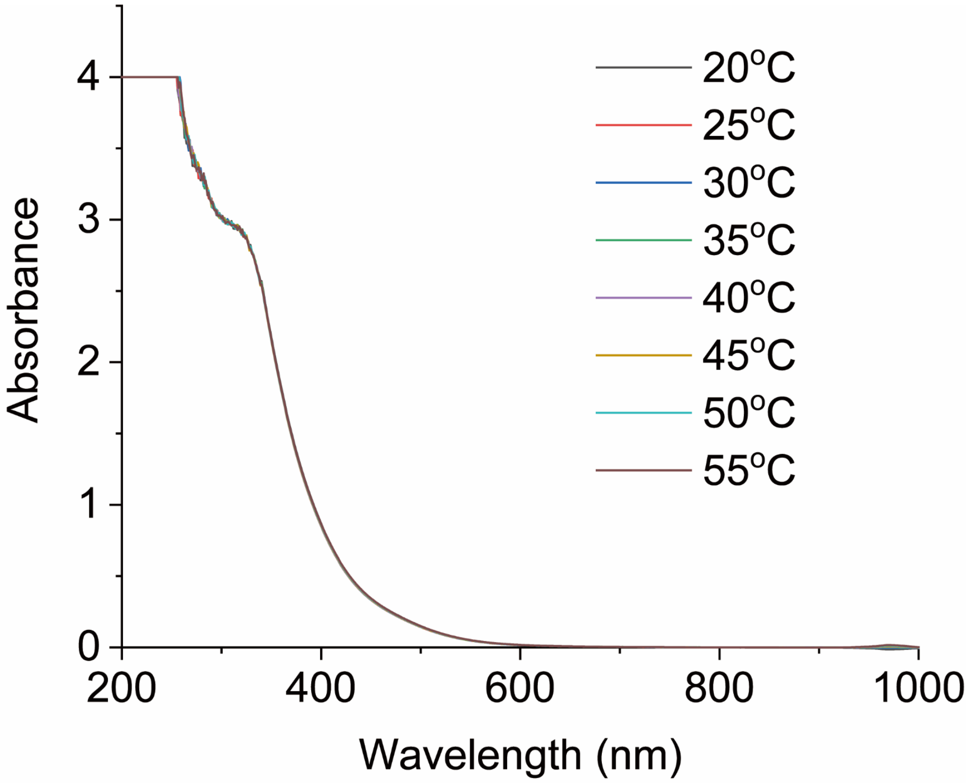

Figure S2. The absorption spectra of Au-AgNCs@BSA at different temperatures.

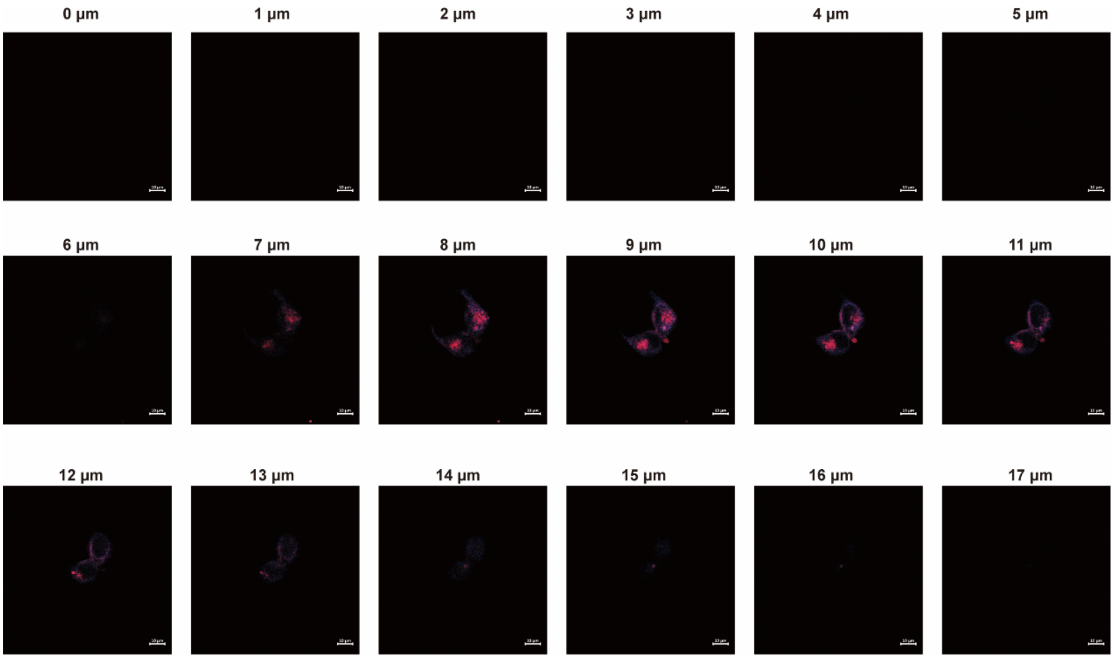

Figure S3. The fluorescence microscopy imaging of HeLa cells, pretreated with Au-AgNCs@BSA. Images captured at different Z-positions demonstrate that the nanoparticles are efficiently internalized by the cells.
